# Autologous Platelet-Rich Plasma Enhances the Healing of Large Cutaneous Wounds in Dogs

**DOI:** 10.3389/fvets.2020.575449

**Published:** 2020-10-26

**Authors:** Ilaria Iacopetti, Marco Patruno, Luca Melotti, Tiziana Martinello, Silvia Bedin, Tamara Badon, Edoardo Maria Righetto, Anna Perazzi

**Affiliations:** ^1^Department of Animal Medicine, Production and Health, University of Padua, Padua, Italy; ^2^Department of Comparative Biomedicine and Food Science, University of Padua, Padua, Italy; ^3^Department of Veterinary Medicine, University of Bari Aldo Moro, Bari, Italy; ^4^Veterinary Practitioner, Padua, Italy

**Keywords:** dog, autologous platelet-rich plasma, regenerative medicine, subacute wounds, cutaneous wound healing

## Abstract

Platelet-rich plasma (PRP) is known to play a crucial role in skin wound healing, in both Human and Veterinary Medicine. Remarkably, until now, no studies have reported PRP treatment in subacute full-thickness skin wounds of the dog. The aim of this study was to evaluate the effects of two consecutive applications of autologous PRP, with the second application after 15 days, in 6 dogs showing large subacute skin wounds. The percentage of contraction, re-epithelialization and healing in all treated patients indicated that no complications or side effects, associated with consecutive PRP treatments, occurred in any patient and all wounds achieved complete closure and re-epithelialization. Our results suggest a positive effect of repeated autologous topical PRP treatments in large cutaneous subacute wounds of different etiology. Therefore, this PRP treatment could represent a simple, cost-effective, and valid alternative to promote healing processes in subacute large wounds cases in dogs.

## Introduction

Cutaneous wound healing is a physiological process that is triggered after the loss of skin integrity (1–3). This complex and dynamic response to a physical trauma comprises three overlapping phases (inflammation, proliferation and remodeling) that are initiated and orchestrated by a cascade of local responses elicited by tissue injury (2, 4–6). In particular, during the inflammatory phase, which reaches its peak in 2–3 days, an active role is played by platelets that release different growth factors and cytokines promoting inflammatory cells recruitment and activation. The proliferative phase involves the deposition of newly formed extracellular matrix (ECM) (collagen I fibers), epithelialization and angiogenesis stimulation reaching its climax between 2 and 3 weeks after injury. The remodeling phase, less rapid and reactive, starts between 2 and 3 weeks following injury leading to the reorganization of the collagen matrix and replacement of the granulation tissue with an acellular scar (7, 8). Complete wound healing depends on several parameters such as etiology of the lesion, position in the body, blood supply, defect size, tension and mobility of wound margins, susceptibility to infection and type and, in the case of large wounds, condition of the underlying tissue (9–11).

On the basis of the time in which the wound is created, cutaneous wounds can be divided into three types: (i) acute wounds, of recent formation, (ii) subacute wounds starting from about 1 week after injury, and (iii) chronic wounds if there is no healing within 6 weeks (12, 13). A delay of normal reparative processes, for different reasons, can cause subacute and chronic wounds that lead to an extension of the therapies and greater economic costs for the healing of the patients.

Platelet-rich plasma (PRP) is known to enhance wound healing and tissue regeneration, given the high number of platelets, growth factors and cytokines in it contains (9, 14, 15); this product represents a natural physiological mixture of stimulatory and inhibitory mediators that have synergistic biological effects in the wound healing environment (16). Platelets secrete several growth factors and active metabolites, and their use can have a positive influence in clinical situations requiring rapid healing and tissue regeneration considering that they are also involved in mesenchymal cell recruitment and ECM synthesis (14, 17–19). Indeed, wounds treated with PRP present an increased re-epithelialization, contraction, and neovascularization (2).

In human medicine, topical application of growth factors derived from platelets encouraged the repair of previously non-healing wounds (20, 21). The beneficial therapeutic effects of PRP, with its adhesive, hemostatic and healing properties on tissue regeneration, are widely recognized in literature as an useful treatment for both cutaneous acute wounds and chronic non-healing ulcers in patients (9, 22–25).

Positive results have been obtained also in the veterinary field, both in acute wound experimental models (26) or in the clinical practice to treat chronic non-healing wounds. In the literature, there are various reports on the use of PRP in veterinary medicine demonstrating that it might accelerate and stimulate the healing of acute wounds and promote the repair of impaired chronic wounds as observed in dogs (12, 27–32), horses (1, 16, 33–36), cats (37, 38), sheep (39), goat (40), and pigs (41, 42). However, sometimes these results appear to be discordant, i.e., no positive effects of PRP are shown (2, 43, 44).

The aim of the present study was to describe the wound management and the effects of the topical application of PRP, used in two consecutive applications with the second application after 15 days, in large subacute skin wounds in dogs, occurred 1–2 weeks before the first treatment.

## Materials and Methods

### Ethics Statement

The study was carried out with client-owned dogs. All animal owners included in the study signed a written consent after having been notified of the relevant project information. The informed consent was discussed during the consultation and contained information about the properties of the treatment and post treatment instructions. All dogs that participated in the study were directly overseen by a veterinarian to ensure no harm was incurred during study participation.

### Inclusion Criteria

The current study was conducted in the Veterinary Teaching Hospital of University of Padua and performed by the same team. To be included in the study, dogs of any breed, gender and age, had to present full-thickness skin defects from 1 to 2 weeks, in order to evaluate the wounds as subacute. The defects in different regions of thorax and abdomen were due to different etiological causes. Dogs were excluded if surgical treatment was applicable.

All dogs were selected with defects in different regions of the body and due to different etiological causes.

### PRP Preparation and Application Protocol

Basic hematological and biochemical profiles were performed in all cases before PRP preparation to avoid the presence of other concomitant pathologies. Autologous PRP was prepared from peripheral venous blood with a centrifuge (Labofuge 400, Heraeus Holding, Hanau, Germany) using a double centrifugation tube method (first at 2,800 rpm for 20 min and then at 1,300 rpm for 15 min) described by Perazzi et al. (45). A range of 15–25 mL volume of blood (matched to patient weight and wound size) was collected with commercially designed platelet sequestration tubes containing sodium citrate (Vacutainer CPT; Becton, Dickinson and Company, Franklin Lakes, New Jersey, USA) and then centrifuged to obtain liquid PRP. The final volume of PRP contained a mean ± SD concentration of 1,259, 50 × 10^3^ ± 175,50 platelets/mL, 4- up to 6-folds the initial platelets concentration. After a wound bed preparation, depending on the size of the different lesions, in awake patients a total of 3–6 ml of PRP was dropped on the wound surface including on the edge of the wound to fully cover the area of the lesion. For wounds <50 cm^2^ a total volume of 3 ml of PRP was applied, for wounds between 50 and 80 cm^2^ 4 ml, for wounds larger than 80 cm^2^ 6 ml. When detachment of the underlying soft tissue was present, 1 ml of PRP was reserved and applied in the depth of the cavity. In all cases the wounds were protected by a light dry to dry covering bandage changed after 3 days the first time and subsequently weekly, after saline cleaning. Amoxicillin-clavulanic acid treatment (Synulox, Pfizer A.H., New York, USA) at a dose of 20 mg/kg twice daily *per os*, was administered for 7 days; no NSAID therapy nor other local treatments were administered to avoid interference with the mechanism of action of the PRP. Elizabethan collars were always applied. PRP topical application was repeated 15 days after the first treatment. The management of the second treatment was similar to the first application.

### Data Evaluation and Image Analyses

Wounds were documented with photographs to measure wound area using a ruler as a reference measure. The images were captured before treatment, 3 days after the first PRP treatment, and weekly; the dimensions of the wounds, expressed in cm^2^, were determined using ImageJ software (Rasband, W.S., ImageJ, U. S. National Institutes of Health, Bethesda, Maryland, USA, http://imagej.nih.gov/ij/, 1997–2012), with specific plugins. The value obtained represents the average calculated from 3 repeated measurements performed for each image at the different timings to reduce the operator error. Percentage of the wound contraction, re-epithelialization and wound healing, were calculated for each wound based on the formulas already widely reported in the literature by many authors (46–49). Percentage of wound contraction = 100-(A_tx_/A_t0_^*^100). where A is the area/size that has as its perimeter the margin of the initial lesion; t0 = initial wound size; tx is wound size at day analyzed. Percentage of wound re-epithelialization = Aw_tx_/A_tx_^*^100; where Aw_tx_ is, the size of the area of epithelialization at the day analyzed and A_tx_ is the size of the area of wound at the day analyzed. Percentage of wound healing = 100- percentage of non-healed area compared to the wound size at T0 = wound healing area; where percent of non-healed area = area of granulation tissue at day X/initial size of wound (T0) ^*^ 100.

## Results

Six dogs were enrolled in this study. Three dogs were purebred (1 Saint Bernard, 1 Bracco and 1 Segugio) and three dogs were mixed-breed. There were 3 male and 3 female. The age ranged between 11 months and 11 years. Body weight ranged between 8 and 42 Kg.

All the lesions treated had occurred for different reasons 7 to 15 days before treatment and were localized in different parts of the body (skull, thorax, dorsal and inguinal region). The treated wounds measured between 11.32 and 91.5 cm^2^. Patients reporting data, etiology, position, onset time, dimensions of the wounds, PRP used and time for healing are summarized in Table 1. Debridement and wound bed preparation were performed in all cases before the first treatment only; subsequently, it was not necessary for the second treatment.

**Table 1:** Patient characteristics, wound size before treatment, onset time, platelet concentration, number and amount of treatment and healing time.

**Case**	**Breed**	**Sex**	**Age**	**Weight (kg)**	**Etiology**	**Body position**	**Onset time (days)**	**Initial skin wound (cm^**2**^)**	**Platelet concentration × 10^**3**^ platelets/μL**	**PRP used 1° time (ml)**	**PRP used 2° time (ml)**	**Time for healing (days)**
1	Saint Bernard	F	11 m	42	Suture dehiscence for Surgery asportation	Left fronto-occipital portion of the skull up to right ear	7	18,76	1,550	3	2	40
2	Mixed breed	F	11 y	25	Bite by another dog	Dorso-lateral region right side	7	47,89	1,235	2+1	1+1	30
3	Bracco	F	6 y	30	Suture dehiscence for hunting accident	Right thorax, axillar and pectoral region	15	79,98	1,259	6	4	45
4	Mixed breed	M	10 y	8	Burn lesion	Dorsal region	15	91,5	1,050	6	4	Not note
5	Segugio	M	8 y	12	Dog fight	Dorsal and right lateral thorax	10	77,06	1,123	4	3	40
6	Mixed breed	M	4 y	15	Dog fight	Inguinal right region	15	11,32	1,340	2+1	1+1	30

**Figure 1:**
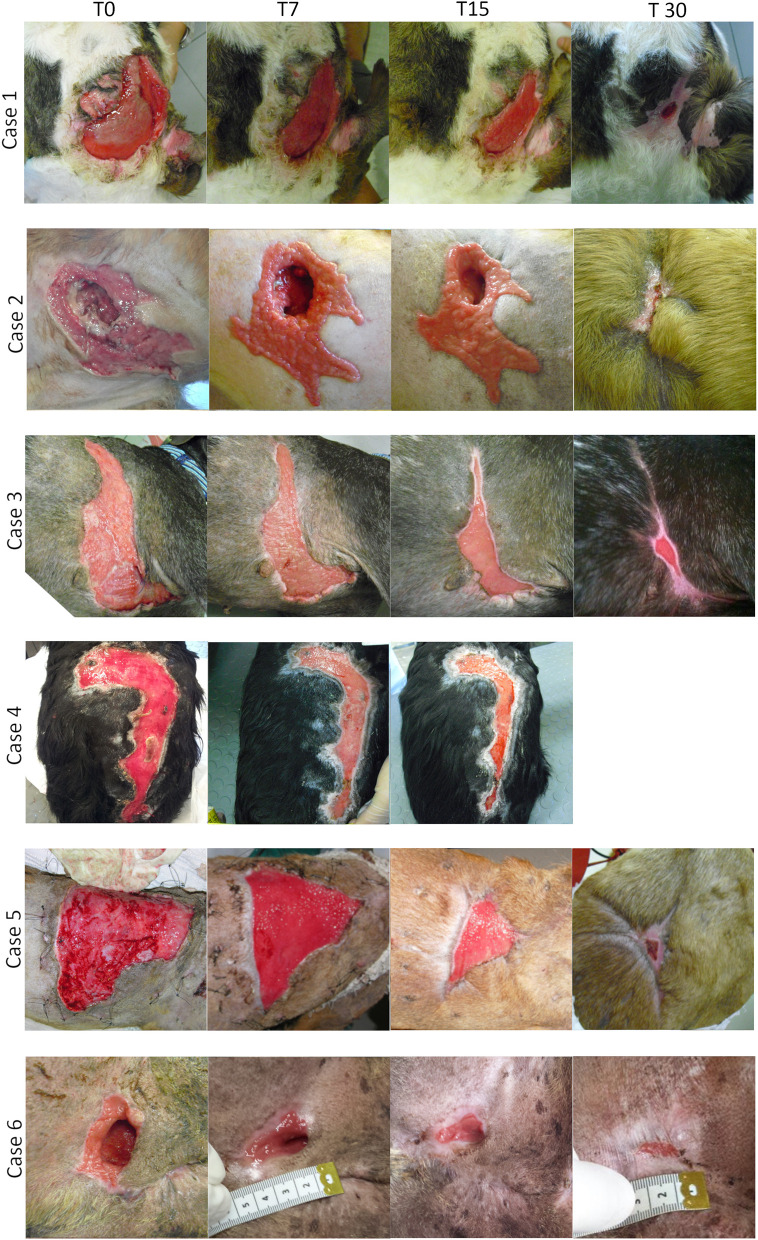
Serial macroscopic images of the wound site of the six case at different time points (T0, T7, T15, T30). Treatments were applied after T0 e after T15. Case 4 was missing at the final 30 day follow up.

Figure 1 shows the different stages of wound healing in all patients treated from the first PRP (day 0) treatment up to 30 days of follow up. Unfortunately, case 4 was missing at the final 30 day follow up visit. In all patients from the first post-treatment control (3 days after the application), lesions became exudative and, within 2 weeks, the amount of effusion decreased significantly. The necrotic tissue, when present, gradually disappeared and a considerable amount of granulation tissue progressively covered the bed lesion. Systemic antibiotic therapy was administered for only 7 days post treatment, and in no case there were signs of systemic infection, even where it was infected lesions as in the case 2, 5, and 6. In cases 2 and 6, since a detachment of underlying soft tissue was present, 1 ml of PRP was inserted deep into the cavity. No adverse reactions or side effects were observed, even in cases where PRP treatment was repeated in high doses. Wounds were considered healed when they were completely covered by the epithelium. All wounds achieved complete closure and re-epithelialization, and no complications associated with PRP treatment occurred in any patient. Healing time varied between 30 and 45 days except in case 4, where the dog missed the 30 day follow up.

**Figure 2:**
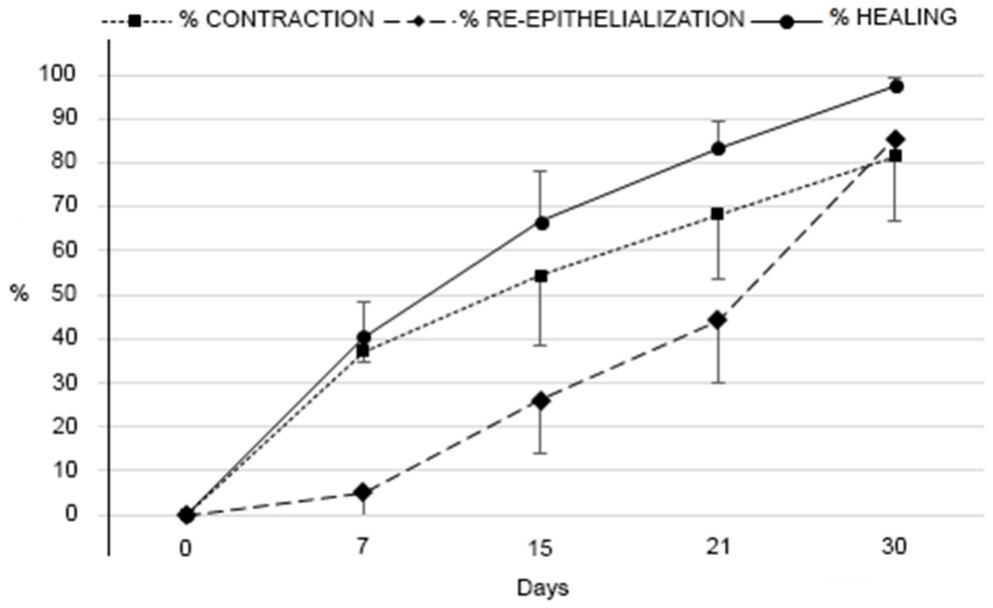
Percent of wound contraction, wound re-epithelialization, and wound healing measured weekly.

No abnormal tissue formation, keloid or pathologic scarring were observed. In all cases, a hair regrowth occurred even where large losses of skin were present. Beneficial effects in large delayed healing skin wounds seem to have occurred in all treated cases. In Figure 2 are reported the percentage average of wound contraction, wound re-epithelialization and wound healing measured weekly and treated with 2 applications (at T0 and T15) compared to the wound size at T0 before first treatment.

In particular, data showed that the contraction increased quickly after the first treatment, the percentage change between T0 and T15 was 57.16%, and after the second treatment continued to increase linearly, the percentage change between T15 and T30 was 24.28%. Re-epithelialization, with respect to the contraction trend, presented an exponential growth, where the values increased in proportion to its previous value, almost doubling. In fact, the percentage change between T0 and T15 was 26.04%, between T15 and T30 was 59.57%. Considering the percentage average of healing, data showed that the first treatment led to a percentage change (T0–T15) of 66.73%, while the second treatment induced a percentage change (T15–T30) of only 30.77%.

## Discussion

Large cutaneous wounds are an economical and physiological burden both in human and veterinary medicine because they require long healing times. Different innovative treatments have been tested during the years and, among them, PRP stands out for its beneficial characteristics. PRP is a readily available and cost-effective therapy rich in growth factors and cytokines that enhances and supports tissue regeneration by stimulating cell migration and proliferation. Its application for the treatment of skin ulcers has already been widely reported both in humans (9, 22–25) and animals (1, 16, 29, 37, 39, 42). However, studies regarding the clinical efficacy of PRP in dogs for skin wound healing are rare. Particularly, in experimental acute full-thickness wounds trial, different studies reported a positive outcome with a better quality of the newly regenerated skin respect than the control group after a single administration of locally injected autologous PRP treatment (28, 32) and in sub-dermal plexus skin flap (50). Moreover, beneficial effects are also reported after repeated injections as three (30) or four times (31).

In contrast others have shown that the topical application of PRP in canine medicine for skin wound healing did not lead to any amelioration of the healing process compared to control groups or other treatments (2, 43, 44). These discrepancies might be due to variables between experiments such us experimental wound model, PRP biology, preparation techniques and differences in treatments protocol or time-points investigation (43).

Only few clinical papers report treatments in chronic or non-healing skin wounds in dogs; furthermore every study reported PRP treatments with a single topic application (12, 27, 29). Tambella et al. (12) described topical application of autologous platelet gel (PLG) to treat chronic decubital ulcers in 18 dogs showing more rapid healing in lesion treated with PLG compared to control wounds. In the other two reports, the beneficial effects with improvement in the management of chronic delayed wound healing were described in a single clinical case (27, 29). Finally, there are no reports in the literature describing the treatment of subacute skin lesions using PRP in dog.

This is the first report describing a two consecutive topical application of autologous PRP for the treatment of large subacute skin wounds on a series of dogs. Six dogs presenting a subacute large skin wound (wound area, mean ± SD 54.42 ± 13.8 cm^2^), which occurred between 1 and 2 weeks before enrolment, were included in the present study. All wounds, of different size, etiology and position in the body, were treated with a topical application of autologous PRP and it was repeated after 2 weeks. The decision to apply PRP after 2 weeks after the first treatment was based on the fact that the biological half-life of the platelets in dogs, as in human, is about 10 days; this way wound healing was stimulated for a second time after the physiological platelet apoptosis (51, 52). The second application of topical autologous PRP seems to highlight an improvement in contraction and re-epithelialization in all wounds. Particularly, a faster wound contraction rate was observed during the 15 days after the first treatment and showed a continuing increase following the second treatment. In contrast, re-epithelialization showed better improvement after the second treatment. These clinical data could indicate that repeat the PRP application after 15 days can be useful for both contraction and re-epithelialisation of the wound by supporting their clinical use also in subacute skin wounds as well as observed in experimental studies for acute skin wound healing in dogs (30, 31).

All wounds healed completely between 45 days after the first PRP treatment and no abnormal tissue formation, keloid or pathologic scarring was observed after this protocol of PRP treatment similar to that reported also by Tambella at al. (12). Moreover, a full hair regrowth was observed in all patients as reported also in the literature (27, 29). In all cases, cleaning was only carrier out before first PRP application. Antibiotics were administered for only 1 week after the first treatment but no evidences of wound infection were observed until complete healing in all patients. This might be due to the antibacterial properties of PRP that have may contributed to the prevention of infection (19, 31). NSAID were not administered after PRP treatments because they could interfere with platelets activation and aggregation, thus impairing its beneficial effects on wound healing (53–57). After PRP treatments, all lesion were covered by a light bandage, these bandages were changed first after about 3 days to verify the wound conditions then later weekly. This decision was made to avoid removing the newly formed tissue by changing the bandage. No tissue damage was observed upon dressing removal using abundant saline solution; similar data are reported by Tambella et al. (12).

To the Authors knowledge there are no published data describing the effect of two consecutive applications, with the second application after 15 days, in large subacute skin lesions in dog. Moreover, no repeated PRP protocols are reported in the canine medical field. Despite the observed beneficial effects and considering that it is the only study performed on subacute lesions the comparison of our data with other studies reported in the literature is challenging since the lack of control lesions and the different initial dimensions of the lesions.

The limitations of the present case series study include the low number of sample, the heterogeneity of the wounds types and the absence of control wounds, since the clinical nature of the study. Further studies using a larger number of animals (eventually experimental animals) are required to validate the results obtained and to point out a clear mechanism of action.

In conclusion, our results suggest that a topical application of autologous PRP, repeated once with a time gap of 2 weeks, may represent a simple, safe and cost-effective therapy for subacute skin wounds in dogs. No complications associated with our protocol of PRP treatment occurred in any patient and all wounds achieved complete closure and epithelialization. The PRP could be useful to enhance wound repair representing a valid alternative therapy to promote cutaneous wound healing in subacute large skin wounds when traditional surgery cannot be performed for different reasons.

## Data Availability Statement

The raw data supporting the conclusions of this article will be made available by the authors, without undue reservation.

## Ethics Statement

Ethical review and approval was not required for the animal study because the current clinical study was carried out with client-owned dogs. All animal owners included in the study signed a written consent after having been notified of the relevant project information. The informed consent was discussed during the consultation and contained information about the properties of the treatment and post treatment instructions. All dogs that participated in the study were directly overseen by a veterinarian to ensure no harm was incurred during study participation. An ethical review process by the Animal Welfare Committee of the University of Padua was not required for our study. PRP's safety has been established for over 20 years for its wound healing properties and it's proven effectiveness has extended across multiple medical specialties both in Human and Veterinary Medicine, including orthopedics, sports medicine, dental and maxillofacial surgery dermatology (chronic wound healing), and ophthalmology, cosmetic surgery. PRP is safe, tested and effective because it is autologous enriched plasma. Written informed consent was obtained from the owners for the participation of their animals in this study.

## Author Contributions

ER, TM, and LM: acquisition of data and analysis and interpretation of data. AP and II: contributions to conception and design and acquisition of clinical data. SB and TB: contributions to laboratory test. II and MP: drafting the manuscript and revising. All authors read and approved the final manuscript.

## Conflict of Interest

The authors declare that the research was conducted in the absence of any commercial or financial relationships that could be construed as a potential conflict of interest.
